# Impact of Early Oral Feeding on Nasogastric Tube Reinsertion After Elective Colorectal Surgery: A Systematic Review and Meta-Analysis

**DOI:** 10.3389/fsurg.2022.807811

**Published:** 2022-03-22

**Authors:** Yan Wang, Yanji Zhang, Xing Hu, Hui Wu, Shan Liang, Jing Jin, Yunjun Wu, Yao Cen, Zairong Wei, Dali Wang

**Affiliations:** ^1^Sichuan Cancer Hospital, Chengdu, China; ^2^The Affiliated Hospital of Zunyi Medical University, Zunyi, China; ^3^Zunyi Medical University, Zunyi, China

**Keywords:** early oral feeding, elective colorectal surgery, nasogastric tube reinsertion, systematic review, meta-analysis

## Abstract

**Background:**

Colorectal cancer is a common malignant tumor appearing in the gastrointestinal tract. Surgical resection is recognized as the best means to improve patient survival. However, it is controversial whether early oral feeding (EOF) after elective colorectal resection demonstrates safety and efficacy in concerned clinical outcomes.

**Methods:**

We searched PubMed, Embase, Cochrane Library, and CNKI from inception to September 2021. Two authors independently screened the retrieved records and extracted data. EOF was defined as feeding within 24 h after surgery, while traditional oral feeding (TOF) was defined as feeding that started after the gastrointestinal flatus or ileus was resolved. The primary outcome was nasogastric tube insertion, and the secondary outcomes were the length of hospital stay and total complications. Categorical data were combined using odds ratio (OR), and continuous data were combined using mean difference (MD).

**Results:**

We screened 10 studies from 34 records after full-text reading, with 1,199 patients included in the analysis. Nasogastric tube reinsertion (OR 1.69; 95% CI 1.08 to 2.64, *p*=0.02) was more frequent in the EOF group, and older ages (>60 years) were associated with higher risk of nasogastric tube reinsertion (OR 2.05; 95% CI 1.05 to 3.99, *p* = 0.04). Reduced length of hospital stay (MD −1.76; 95% CI −2.32 to −1.21; *p* < 0.01) and the rate of total complications (OR 0.49; 95% CI 0.37 to 0.65, *p* < 0.01) were observed in EOF compared with TOF.

**Conclusions:**

EOF was safe and effective for patients undergoing elective colorectal surgery, but the higher rate of nasogastric tube reinsertion compared with TOF should not be ignored.

## Introduction

Colorectal cancer occupies approximately 10% of all diagnosed cancers, significantly contributing to cancer-related deaths worldwide (1). Although early colorectal cancers could be appropriately managed through endoscopic resection techniques (safer and less expensive than surgery), many patients with confirmed colorectal cancer are still referred for surgery, combined with chemoradiotherapy (2). However, the complications following traditional colorectal cancer surgery occur in 20–30% of cases, with an average postoperative hospital stay of 8–12 days (3).

A multimodal rehabilitation strategy was initiated to reduce the stress of surgery and then developed into Enhanced Recovery After Surgery (ERAS). The ERAS programs have been shown to be safe and beneficial in patients undergoing colectomy, gastrectomy, pancreatic resections, pelvic surgery, etc., and become a standard in preadmission, preoperative, intraoperative, and postoperative periods to not only reduce patients' complications and enhance fast recovery but also save public resources (4, 5). A reasonable feeding protocol is considered an effective way to reduce the length of hospital stay, despite sometimes being identified as a potential factor triggering postoperative complications. In 2017, the ERAS Study Group, which was established in 2001, recommended early intake of oral fluids and solids—a type of early oral feeding (EOF)—to support energy and protein supply and reduce starvation-induced insulin resistance (6).

In past decades, the passage of flatus or bowel movements, which signals the resolution of postoperative ileus, indicates that starting an oral diet is safe. Recent studies, however, question traditional oral feeding (TOF) by indicating that the routine use of a nasogastric tube (NGT) after elective colorectal surgery, which is used in decompression of the gastrointestinal tract and prevention of pulmonary complications, may not be necessary (7–9). With its advantage of improving prognosis without obvious adverse events, EOF was introduced for upper gastrointestinal surgery and rapidly extended to other surgeries (10). However, owing to not meeting the energy target requirement, many of the patients had to receive NGT reinsertion. Therefore, it is necessary to prove the EOF protocol as safe and feasible to implement by clinicians with regards to LOS, postoperative complications, and NGT reinsertion.

The present study aimed to conduct a systematic review and meta-analysis, evaluating the associations between the timing of oral intake and length of hospital stay or postoperative complications after colorectal surgery. Besides, the specific objective was to explore NGT reinsertion by subgroup analysis considering distinct ages.

## Methods

### Selection Criteria

The inclusion and exclusion criteria were determined before performing the study. Studies were considered eligible when: (1) The type of feeding was oral, in which EOF was defined as feeding within 24 h after surgery while TOF was defined as feeding that started after the gastrointestinal flatus or ileus was resolved, (2) the data of NGT reinsertion after elective colorectal surgery were provided, (3) studies were completed before September 2021 with a structured dataset. Studies were excluded for involving a rapid rehabilitation program transcending EOF or TOF. We did not limit the age and sex in these studies as long as there were no severe complications before surgery.

Two reviewers independently screened the eligibility of retrieved articles. Disagreement in study selection was resolved by group discussion and arbitrated by a third reviewer.

### Search Methods

Databases including PubMed, Embase, Cochrane Library, and CNKI were searched from the earliest datasets of each to September 2021. There was no language restriction. Review articles were manually searched to identify additional studies. Article titles and abstracts were screened, and full texts were reviewed independently by two reviewers. The search string used the following keywords and was modified for each: (“colorectal surgery OR colorectal resection” [MeSH]) AND (“oral intake OR oral feeding” [MeSH]) AND (“nasogastric tube reinsertion” [MeSH]).

### Data Extraction and Outcomes

Two reviewers independently reviewed selected studies and extracted data; once discrepancies appeared, reviewers discussed and resolved them through repeatedly referring to the original articles. We attempted to contact the study authors for additional information when any significant information was missed.

Primary outcome was nasogastric tube reinsertion. Secondary outcome measures included: (1) length of hospital stay and (2) total postoperative complications. All outcomes mattered clinically in the context of elective colorectal surgery. We also conducted a subgroup analysis of the data on NGT reinsertion by distinct ages.

### Assessment of Risk of Bias

Two review authors independently evaluated the risk of bias for each study, using the revised risk of bias tool (RoB 2.0). We judged each potential source of bias as high, low, or some concerns, using the criteria for the following domains: (1) randomization process, (2) deviations from intended interventions, (3) missing outcome data, (4) measurement of the outcome, and (5) selection of the reported result.

### Data Analysis

We selected the RevMan 5.3 software from Cochrane Collaboration Network to conduct a meta-analysis. Odds ratio (OR) and weighted mean difference (WMD) were used for dichotomous and continuous outcomes, respectively. Both datasets were presented by a 95% confidence interval (CI). Before the meta-analysis, we evaluated potential heterogeneity among the included studies in two steps. First, we checked whether the studies adopted similar designs by examining the participants included, interventions and controls used, and the outcomes, to ensure that the studies were methodologically and clinically homogeneous. The statistical heterogeneity was explored using *I*^2^ statistics. We recognized *I*^2^<50% as low and *I*^2^>50% as high heterogeneity among the selected studies. The causes of heterogeneity should be analyzed by sensitivity analyses. We conducted a subgroup analysis of patients aged over 60 vs. <60 (referring to the age in EOF) through the mean ages reported in the articles, and another subgroup analysis was performed for major complications vs. minor complications.

## Results

### Characteristics of Studies

**Figure 1:**
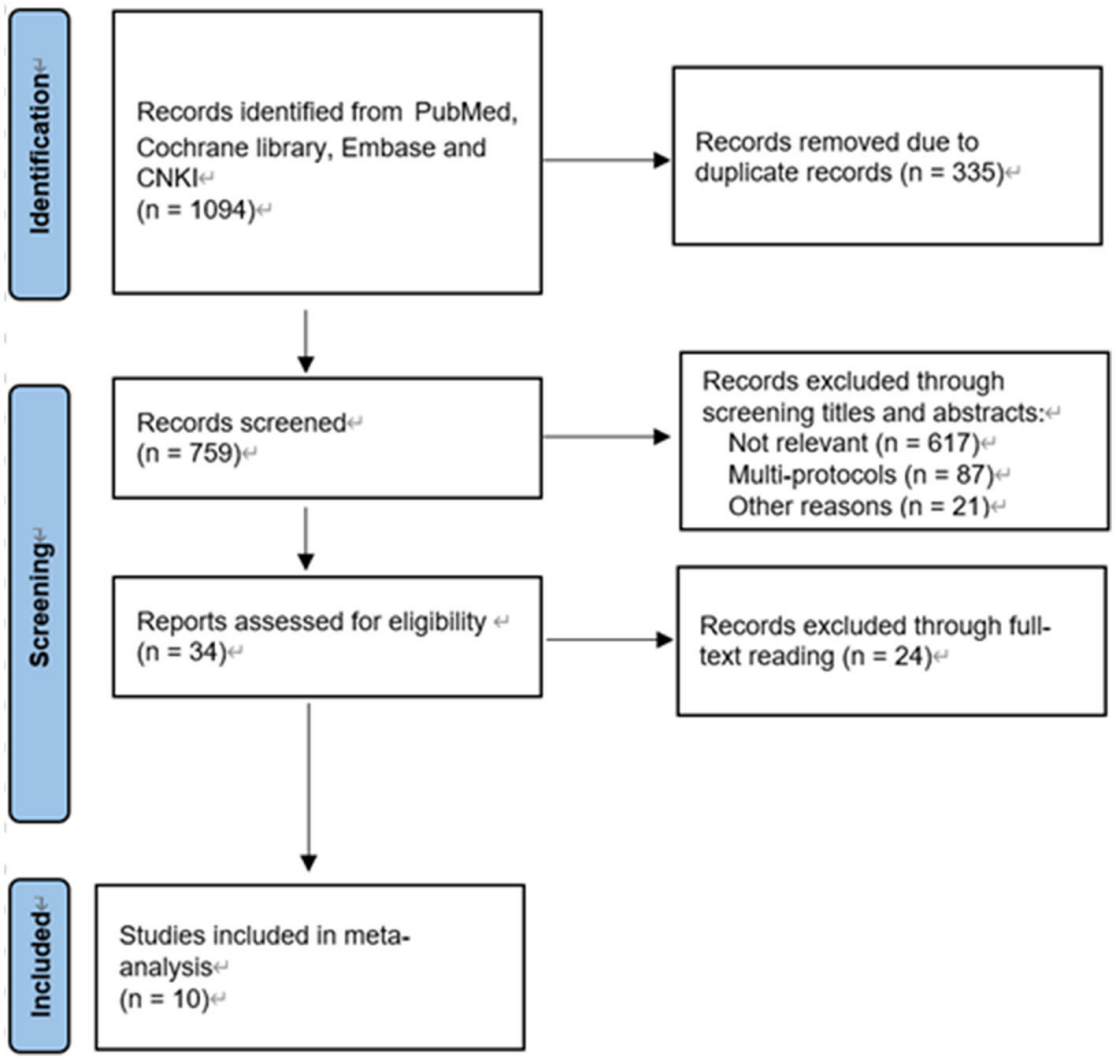
Flow diagram of study selection.

A total of 10 records were identified from PubMed, Cochrane Library, Embase, and CNKI. A total of 759 studies remained after excluding duplicate records. Overall, 34 studies remained after screening titles and abstracts. The remaining 10 studies were screened for quantitative synthesis by reading full texts (Figure 1). There was no limitation in language. Only randomized controlled trials (RCTs) were included. The selected trials included a total of 10 studies and 1,199 patients. Among the included 10 studies from 1995 to 2013, four studies involved patients over 60 years in the EOF group and six <60 years. The site of diagnoses, type of surgery, feeding time, age, and gender are listed in Table 1. Five studies were classified as low risk of bias, and the other five studies were classified with some concerns (Supplementary Figure 1).

**Table 1:** Characteristics of the included studies.

**Study**	**Year of**	**Country**	**diagnosis**	**Feeding time**	**EOF**	**EOF**	**EOF number**	**TOF**	**EOF**	**EOF number**
	**publication**			**of TOF**	**cases**	**age (mean)**	**of males**	**cases**	**age (mean)**	**of males**
Chen et al. (11)	2010	China	100% malignant	UPOF	160	61.2	92	160	58.3	95
Dag et al. (12)	2011	Turkey	100% malignant	UPOF	99	62	52	100	61	61
Feo et al. (13)	2004	Italy	100% malignant	UPOF	50	67.6	NR	50	67.6	NR
Hartsel et al. (14)	1997	America	64% malignant	AROI	29	66	NR	29	68	NR
Lucha et al. (15)	1997	America	Not reported	AROI	26	51	17	25	51	16
Nakeeb et al. (9)	2009	Egypt	100% malignant	AROI	60	52.3	39	60	56.3	42
Reissman et al. (16)	1995	America	Not reported	AROI	80	51	34	81	56	43
Stewart et al. (17)	1998	Australia	Not reported	UPOF	40	58	19	40	59	18
Wang et al. (18)	2013	China	100% malignant	UPOF	24	56.3	20	24	54.3	13
Yang et al. (19)	2010	China	100% malignant	UPOF	32	57.2	20	30	59.5	23

### Description of Results

#### NGT Reinsertion

**Figure 2:**
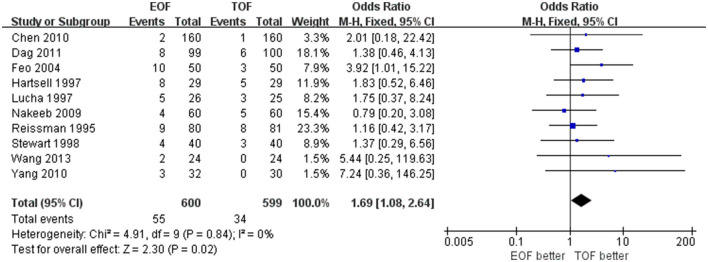
Forest plots of nasogastric reinsertion. All of the included studies were synthesized.

All data in the selected studies were presented in forest plots. We found that the NGT reinsertion rate was higher in the EOF group than in the TOF group (odds ratio [OR] 1.689; 95% confidence interval [CI] 1.08–2.64; *p* = 0.02; *I*^2^ = 0%) (Figure 2). This result showed a significant difference between EOF/TOF and NGT reinsertion after elective colorectal surgery. In addition, subgroup analysis was performed to explore the different effects of oral intake patterns in distinct ages.

#### Subgroup Analysis

**Figure 3:**
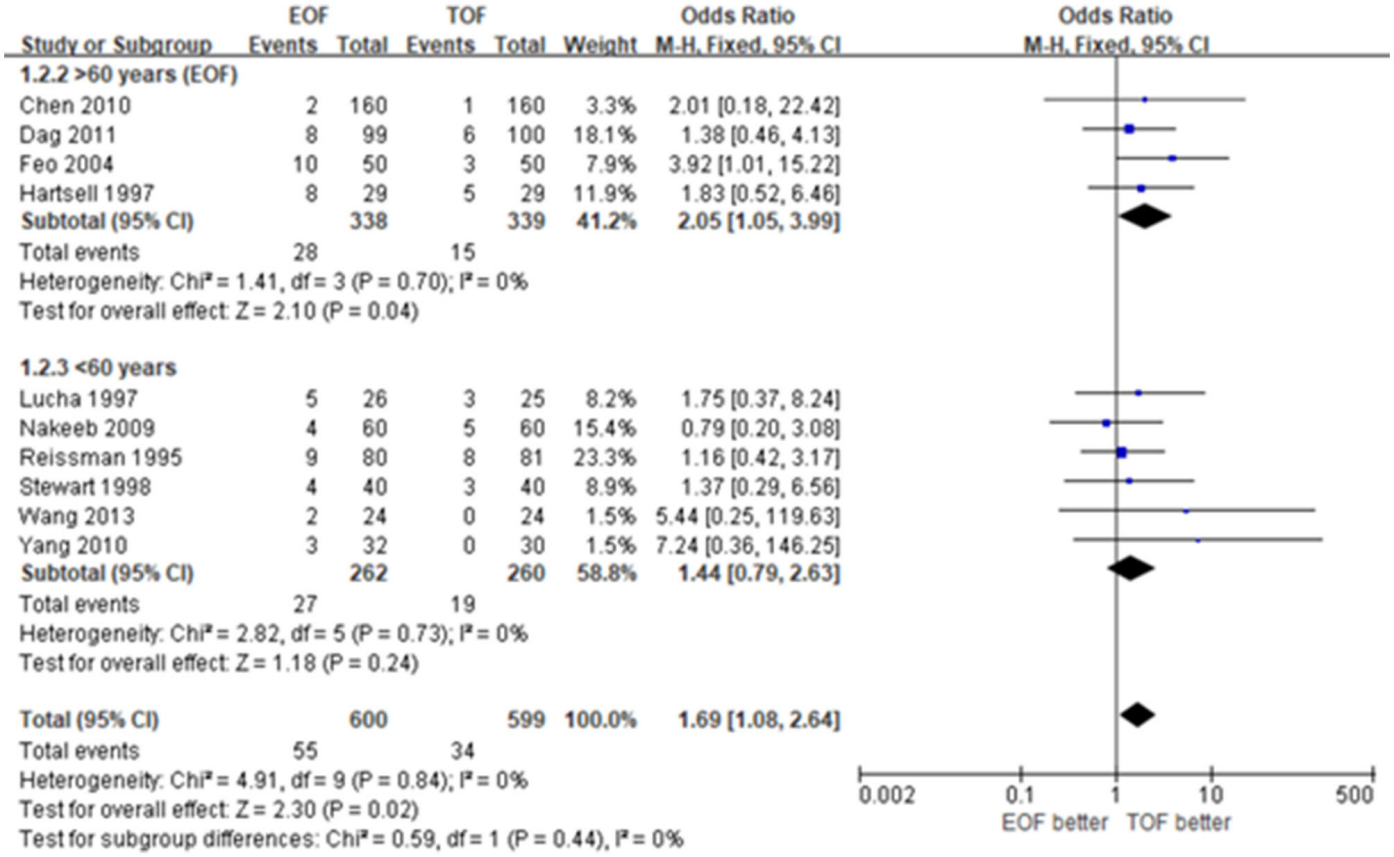
Forest plots of nasogastric reinsertion. **(A)** Patients over 60 years. **(B)** Patients <60 years.

There were four studies involving 677 patients with mean ages over 60 and 522 patients with mean ages <60. EOF was 2.05-fold more likely to be associated with an NGT reinsertion than TOF with low heterogeneity (OR 2.05; 95% CI 1.05 to 3.99; *p*=0.04; *I*^2^ = 0%). No significant difference was found in regard to the NGT reinsertion between EOF and TOF in the group <60 years old (OR 1.44; 95% CI 0.79 to 2.63; *p* = 0.24; *I*^2^ = 0%) (Figure 3).

#### Length of Stay

**Figure 4:**
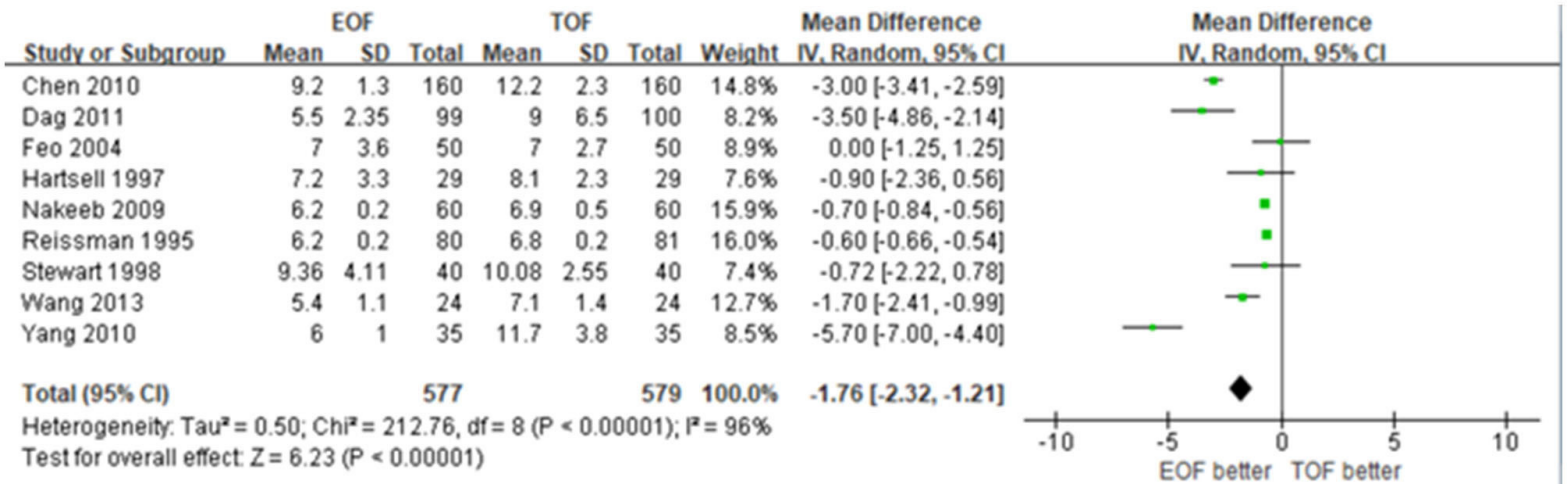
Forest plots of length of hospital stay.

Nine of ten studies provided the data of LOS and demonstrated significant heterogeneity (WMD −1.76; 95% CI −2.32 to −1.21; *p* < 0.01; *I*^2^ = 96%). Given that LOS varied in the included studies, we conducted a sensitivity analysis, presenting that the median LOS was shorter in the EOF group of the studies before 2010 with low heterogeneity (WMD −0.62; 95% CI −0.67 to −0.56; *p* < 0.01; *I*^2^ = 0%) (Figure 4).

### Total Complications

**Figure 5:**
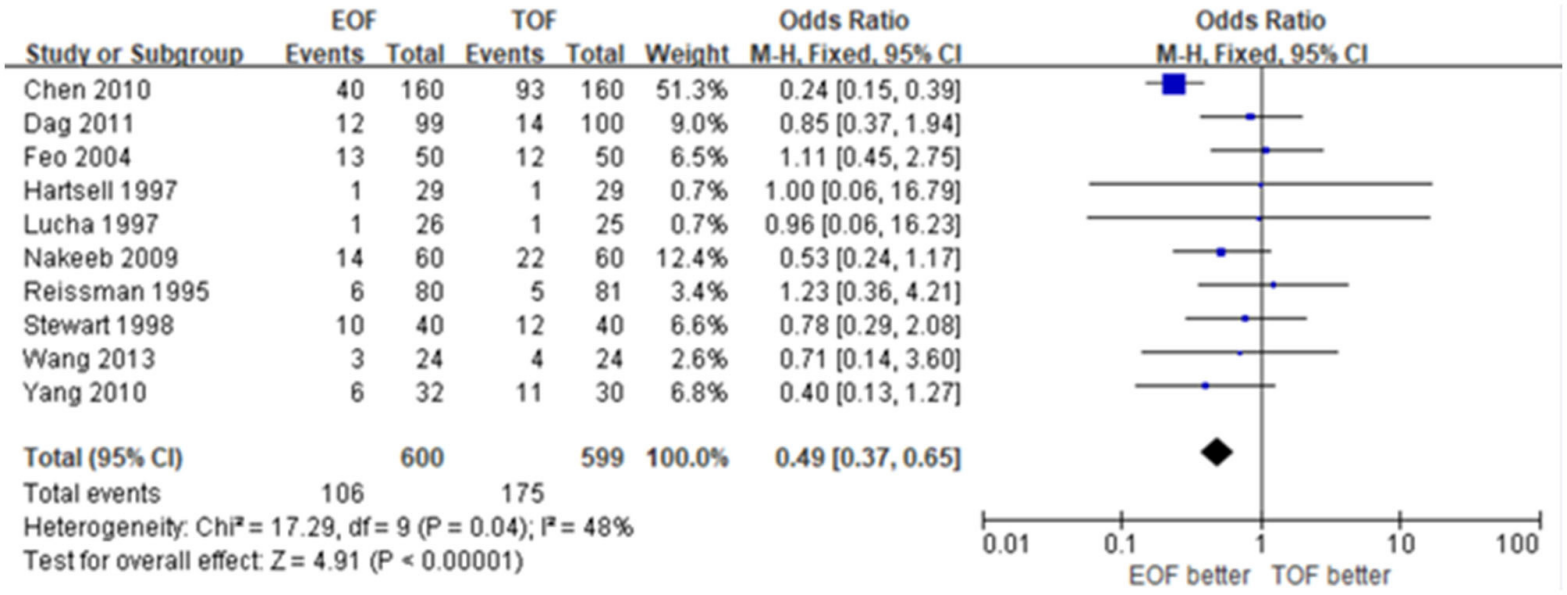
Forest plots of total complications.

**Figure 6:**
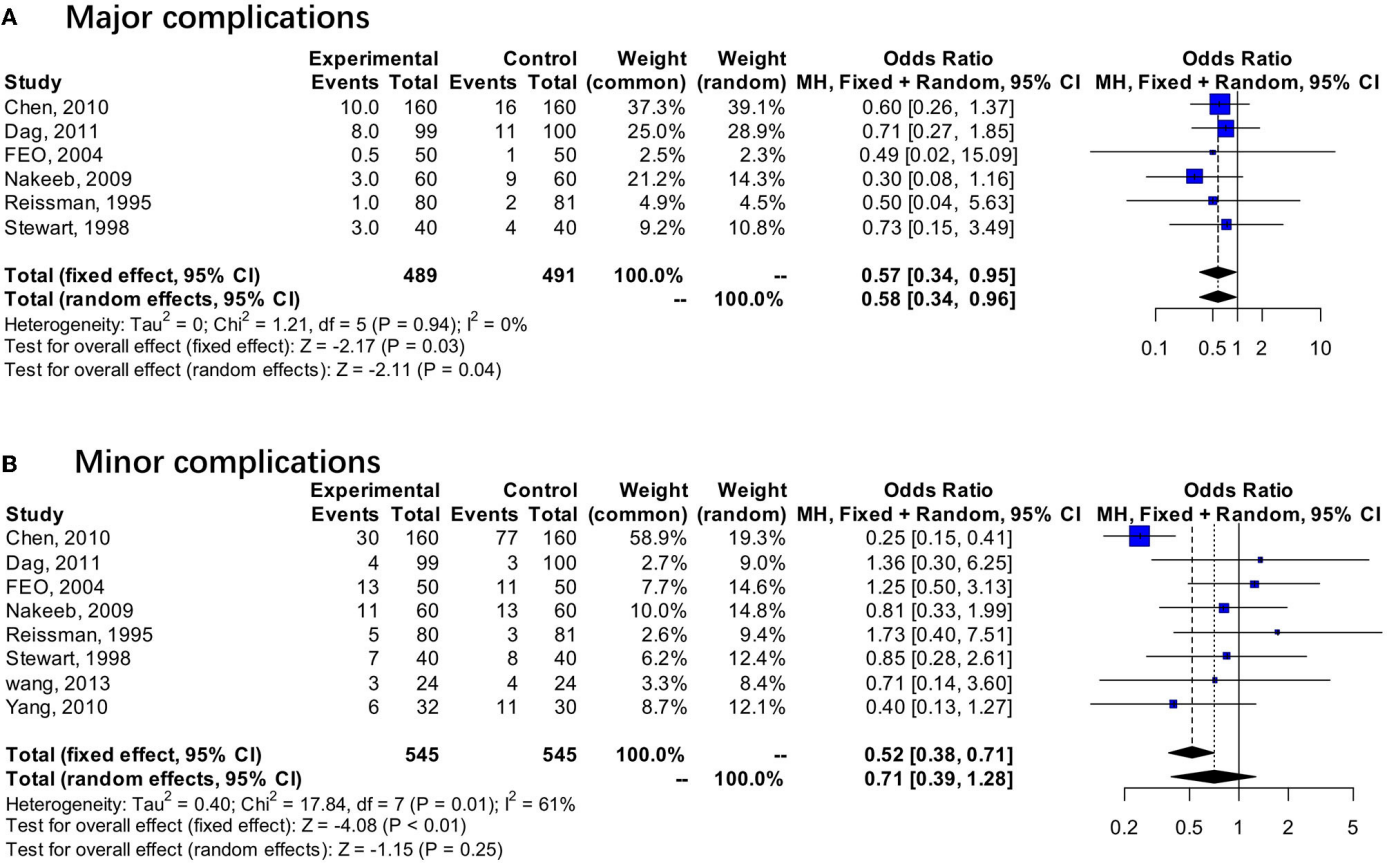
Forest plots of total complications. **(A)** Major complications. **(B)** Minor complications.

All 10 studies accessed the data concerning total complications, which were synthesized in forest plots. There was a significant difference between the EOF group and the TOF group with low heterogeneity (OR 0.49; 95% CI 0.37 to 0.65; *p* < 0.01; *I*^2^ = 48%) (Figure 5). Subgroup analysis showed that EOF was associated with a lower rate of major complications (OR 0.57; 95% CI 0.34 to 0.95; *p* = 0.03; *I*^2^ = 0%) (Figure 6).

## Discussion

Although surgical resection is a primary option to treat colorectal cancer, it also triggers significant postoperative complications and deaths. In traditional postoperative management, patients undergoing colorectal surgery have nasogastric tubes inserted to avoid the oral intake of fluids or nutrients until the postoperative ileus (POI) is resolved. As an important part of the ERAS protocol after colorectal resection, EOF was proposed for postoperative management and presented clear benefits and safety (3, 20). However, it is still controversial whether EOF could improve prognosis without adverse events. Although a recent meta-analysis (21) pooled present clinical trials and provided extensive evidence advocating EOF, the evidence seems inadequate. Many studies other than colorectal surgery were included in the meta-analysis, for example, upper gastrointestinal surgery and small bowel resection.

Admittedly, it was reported that nasogastric tube removal in the immediate course after elective colorectal surgery could improve the rehabilitation of gastrointestinal functions and prevent postoperative infections, thus benefiting patients with shorter LOS and lower postoperative complications (22). Moreover, in a retrospective study involving 1,561 patients (23), the authors suggested that a perioperative strategy with no use of NGT, which could provide a higher tolerance rate of early intake, was proven safe and effective for postoperative rehabilitation. However, some studies emphasized a negative impact on patients' recovery that early feeding exerted. Li conducted a meta-analysis evaluating anastomotic leakage rate after esophagectomy (24). It concluded that the EOF group was more likely to be associated with anastomotic leakage in that type of open surgery. Early feeding without NGT insertion could not trigger any severe complications but postoperative vomiting, for which the surgeon would suspend the EOF protocol (25). These facts indicated that the EOF protocol should be further improved.

In our meta-analysis, we focused on the problem of NGT reinsertion that the EOF protocol may give rise to. On one hand, although the patient accepted EOF at first, surgeons had to reinsert nasogastric tubes and restart tube feeding in response to certain adverse events or according to the patient's requirement. On the other hand, surgeons might apply the strategy of NGT reinsertion once POI has not been resolved within a reasonable period after colorectal surgery, which challenges 25% of the patients (26). Wolthuis proposed NGT reinsertion as the most significant sign of prolonged POI (despite an overestimation of ileus rates), which affects LOS and postoperative complications (27). Therefore, it is inferred that NGT reinsertion is a sign effectively reacting to the patient's recovery. In our study, NGT reinsertion was 1.7-fold more likely to happen in the EOF group than in the TOF group. In the following subgroup analysis, we found that EOF was more associated with NGT reinsertion in the subgroup of older patients. Since NGT reinsertion can make patients uncomfortable and initiate an infection, surgeons should be more conservative about the timing and tolerance of oral intake and more cautious about the EOF protocol for older patients.

However, the results of our study did not infer that EOF should not be performed, instead, we recommended EOF since we observed shorter LOS and lowered complications in the EOF group, consistent with previous findings (28, 29). Shorter LOS and lower complication rates were associated with healthcare expenditures, which was definitely beneficial to patients and healthcare facilities. We assumed that the higher rate in NG tube reinsertion might be correlated with an unmet nutritional requirement, which indicates that the EOF protocol, especially the formula of diets, needs further improvement and more studies.

However, LOS presented a varied result with high heterogeneity, possibly because the surgical method and postoperative recovery have progressed year after year. Therefore, we conducted a sensitivity analysis and found that distinct publications periods may be the source of heterogeneity. The studies published before 2010 presented 0.62 days of LOS shorter in the EOF group with low heterogeneity. Thus, although EOF was deemed safe and effective for feeding nutrition under the ERAS protocol, LOS was inevitably extended once NGT reinsertion or other adverse events happened.

There were several limitations in our study. Firstly, although only RCTs were included in this study, the definition of EOF and TOF, the timing of feeding initiation, and the amount of oral intake differed in selected studies, thus inevitably resulting in bias. Secondly, as mentioned above, NGT reinsertion might be beneficial to relieving the problem of POI, thus trading off the impact of EOF to LOS and complications and finally making the results weaker. Thirdly, the outcome data were not comprehensive enough. Detailed information about NGT reinsertion was missing, e.g., the success rate of NGT reinsertion. These drawbacks are expected to be addressed in well-designed multicenter RCTs in the future. Fourth, we did not use the 2020 PRISMA guideline to guide our study, since the study were performed several steps before we found out that the 2020 PRISMA guideline was released. We believed that the results were not biased by this point, because we conformed to the previous PRISMA version.

All participants received laparotomy, and the EOF feeding time was on the first postoperative day. UPOF refers to the feeding time would not start until passage of flatus. refers the feeding time started after resolution of operative ileus.

## Conclusions

EOF resulted in a high incidence of NGT reinsertion despite a reduction in length of hospital stay and postoperative complications in patients with elective colorectal surgery.

## Author Contributions

YC and XH contribute to selecting the topic. HW and SL contribute to organizing literature and extracting data. JJ contributes to supervising the process and statistics. YWu and YWa contribute to drafting the manuscript. YZ, ZW, and DW contribute to the revision. All authors contributed to the article and approved the submitted version.

## Funding

This study was supported by Guizhou Science and Technology Plan (No. ZK[2021]011) and Provincial and Ministerial Collaborative Innovation Project (No. [2020]39).

## Conflict of Interest

The authors declare that the research was conducted in the absence of any commercial or financial relationships that could be construed as a potential conflict of interest.

## Publisher's Note

All claims expressed in this article are solely those of the authors and do not necessarily represent those of their affiliated organizations, or those of the publisher, the editors and the reviewers. Any product that may be evaluated in this article, or claim that may be made by its manufacturer, is not guaranteed or endorsed by the publisher.
