# Supplementary figures and images for: Impact of Early Oral Feeding on Nasogastric Tube Reinsertion After Elective Colorectal Surgery: A Systematic Review and Meta-Analysis
Source: Front Surg. 2022 Mar 22;9:807811. doi: 10.3389/fsurg.2022.807811 (PMC8980315; doi:10.3389/fsurg.2022.807811)

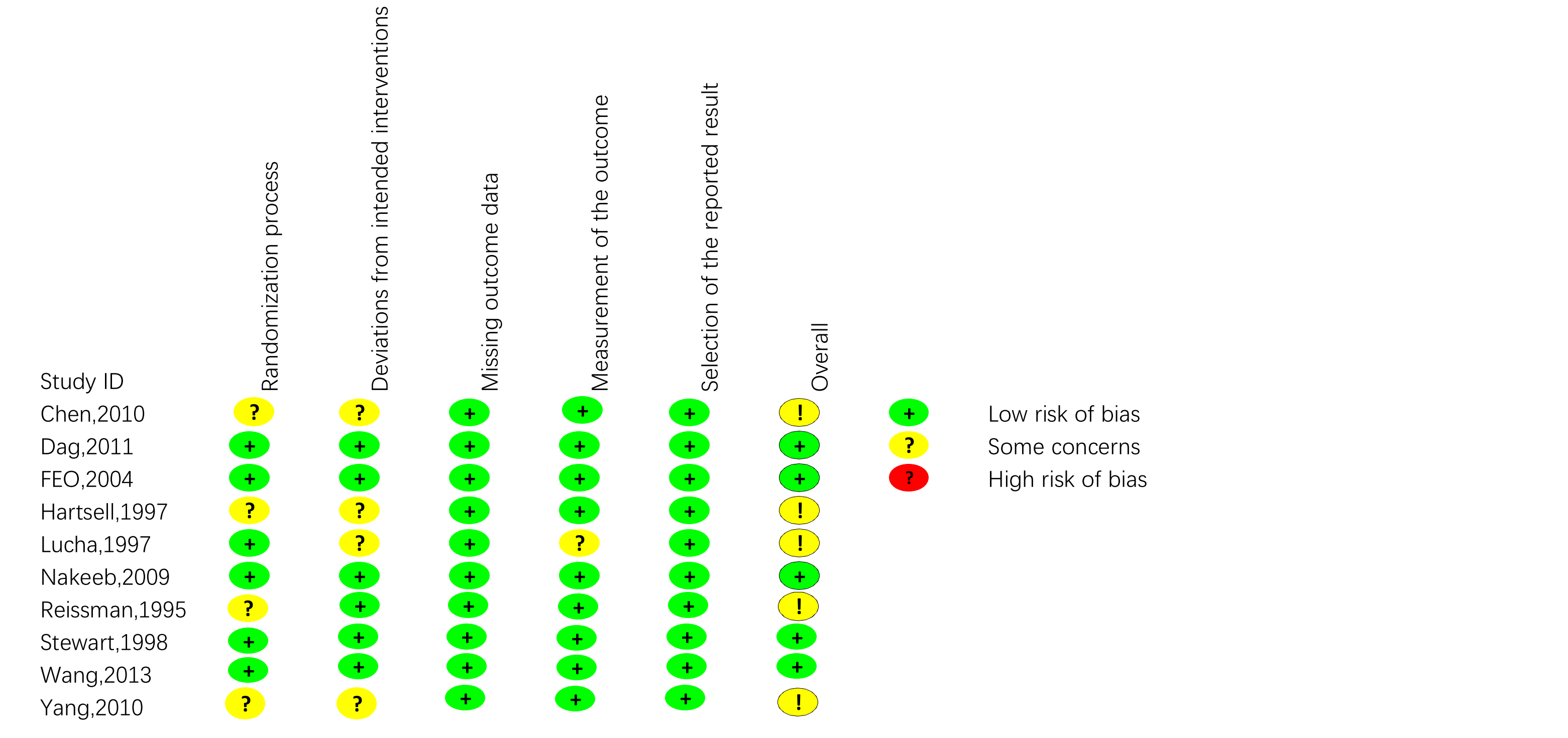

Supplement: Supplementary file 1 [file Image_1.tif]
